# Ni Flower/MXene-Melamine Foam Derived 3D Magnetic/Conductive Networks for Ultra-Efficient Microwave Absorption and Infrared Stealth

**DOI:** 10.1007/s40820-022-00812-w

**Published:** 2022-02-21

**Authors:** Haoran Cheng, Yamin Pan, Xin Wang, Chuntai Liu, Changyu Shen, Dirk W. Schubert, Zhanhu Guo, Xianhu Liu

**Affiliations:** 1 Key Laboratory of Advanced Material Processing & Mold (Ministry of Education), National Engineering Research Center for Advanced Polymer Processing Technology, College of Materials Science and Engineering, Zhengzhou University, Zhengzhou, 450002 People’s Republic of China; 2 Institute of Polymer Materials, Friedrich-Alexander-University Erlangen-Nuremberg, Martensstr. 7, 91058 Erlangen, Germany; 3 Integrated Composites Laboratory (ICL), Department of Chemical & Biomolecular Engineering, University of Tennessee, Knoxville, TN 37996 USA

**Keywords:** Ni-MXene/Melamine foam, Microwave absorption, Heat insulation, Infrared stealth, Flame-retardant

## Abstract

The development of multifunctional and efficient electromagnetic wave absorbing materials is a challenging research hotspot. Here, the magnetized Ni flower/MXene hybrids are successfully assembled on the surface of melamine foam (MF) through electrostatic self-assembly and dip-coating adsorption process, realizing the integration of microwave absorption, infrared stealth, and flame retardant. Remarkably, the Ni/MXene-MF achieves a minimum reflection loss (RL_min_) of − 62.7 dB with a corresponding effective absorption bandwidth (EAB) of 6.24 GHz at 2 mm and an EAB of 6.88 GHz at 1.8 mm. Strong electromagnetic wave absorption is attributed to the three-dimensional magnetic/conductive networks, which provided excellent impedance matching, dielectric loss, magnetic loss, interface polarization, and multiple attenuations. In addition, the Ni/MXene-MF endows low density, excellent heat insulation, infrared stealth, and flame-retardant functions. This work provided a new development strategy for the design of multifunctional and efficient electromagnetic wave absorbing materials.

**Supplementary Information:**

The online version contains supplementary material available at 10.1007/s40820-022-00812-w.

## Highlights

- Ni-MXene/MF foam is synthesized via an electrostatic assembly and dip-coating process.
- The “micro-capacitor” structure of Ni/MXene and the 3D porous structure of MF endow the foam excellent impedance matching and wave absorption performance.
- The excellent heat insulation, infrared stealth, and flame-retardant performances are achieved.

**Supplementary Information:**

The online version contains supplementary material available at 10.1007/s40820-022-00812-w.

## Graphical abstract

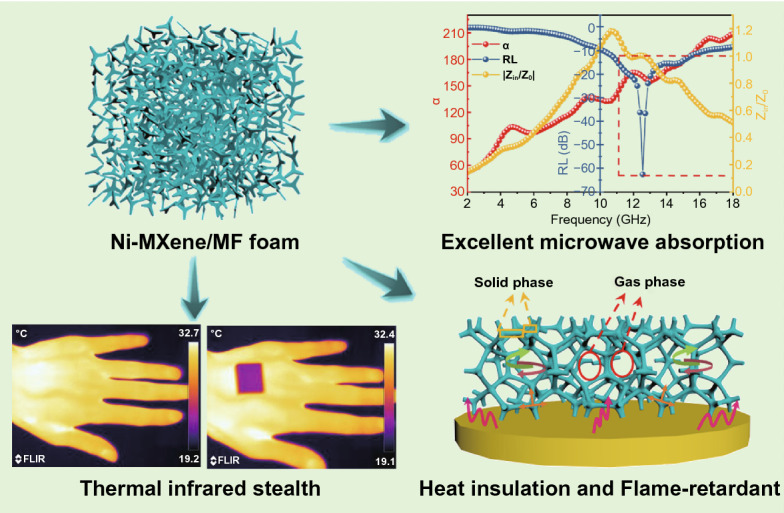

## Introduction

The vigorous development of the 5G era, especially the development of next-generation electronic devices, network communications, and artificial intelligence, has provided high efficiency and convenience for our daily lives while bringing serious electromagnetic (EM) pollution [1–4]. Accordingly, the demand for EM wave absorbing materials has become increasingly urgent. Although there are many traditional EM waves absorbing materials, most of them do not meet the requirements of lightweight, thin matching thickness, wide effective absorption bandwidth (EAB), and strong absorption capacity [5–9]. Therefore, how to effectively absorb EM radiation and meet the requirements of EM wave absorbing materials has attracted widespread attention.

So far, graphene [10], MoS_2_ [11], CNT [12], SiC [13], and MXene have been recognized as representative candidate materials for high-efficiency EM absorbers. The two-dimensional structure of MXene is considered to be the most potential absorbing material due to its strong conduction loss and polarization loss provided by excellent conductivity and abundant surface groups (-OH, -O, -F) [14]. In addition, MXene with a large specific surface area and aspect ratio could be used as an ideal platform carrier to couple with other magnetic components to solve the problem of impedance mismatch caused by its high conductivity [15]. Meanwhile, owing to the abundant functional groups, MXenes could combine with magnetic metal particles and effectively alleviate their stacking and agglomeration while enhancing impedance matching [16]. Liang et al. synthesized Ni/MXene hybrid through a co-solvothermal method, which demonstrated a RL_min_ of -52.6 dB with an EAB of 3.7 GHz at 3 mm [17]. Li et al. fabricated MXene/Ni nanocomposites through the electrostatic self-assembly interaction, which achieved a RL_min_ of -50.5 dB with a narrow EAB at 2.5 mm [18].

Generally, the structure design of absorbing materials improved EM wave attenuation, such as porous, flower-like, core–shell, and hollow structures [19, 20]. Cui et al. obtained porous flower-like MXene/Ni composite microspheres, which show a RL_min_ value of -52.7 dB [21]. Sheng et al. fabricated hierarchical core–shell structure rGO/Ni nanofibers with a RL_min_ of -50.52 dB [22]. Zhu et al. synthesized highly cross-linked 3D structure Fe/Fe_2_O_3_@porous carbon composites with a RL_min_ of -54.7 dB [23]. Gu et al. synthesized hybrid MF, which shows RL_min_ of -59.8 dB with the thickness of 2.3 mm, and EAB of the maximum 5.64 GHz at the thickness of 2.1 mm [24]. Su et al. synthesized SiC_nw_@SiC foam, and achieved a RL_min_ of − 52.49 dB and EAB of 5.6 GHz at a thickness of 2.82 mm [25]. These results indicate that the internal structure of the absorbing material could affect its absorption performance. Among them, the 3D porous structure of MF could endow the absorbing material with the advantages of low density, rich interface polarization, multiple reflections and scattering, thereby attenuating more EM waves [26]. Therefore, combining the highly conductive MXene and magnetic flower-like metal with the 3D porous structure could not only improve the excess electrical conductivity and complex permittivity, and enhance impedance matching, but also provide abundant interfaces and dipole polarization, increase the EM wave multiple reflections and scattering, and is considered a promising strategy to achieve excellent EM absorption property.

To meet the application requirements of the ever-increasing complex environment, advanced absorbing materials must possess multifunctional characteristics in addition to high-efficiency absorbing performance, such as flame retardancy, heat insulation, and infrared stealth, need to be considered [27, 28]. The long-term exposure of the absorbing material to the EM environment will convert the EM wave energy into heat energy, causing local temperature rise, which required the absorbing material to have the requirements of heat insulation and flame retardancy [29, 30]. Furthermore, stealth materials that combine EM wave absorption and infrared shield have broader application prospects in both civilian and military applications [31]. Accordingly, due to the diversity of its actual application environment, it is imperative to develop microwave absorbing materials with thermal insulation, flame-retardant, and infrared stealth.

Herein, we successfully synthesized the two-dimensional hybrids containing flower-like Ni and few-layered MXene through electrostatic self-assembly. Then, the Ni flower/MXene hybrids were assembled on the surface of MF (Ni/MXene-MF) by dipping method, achieving high-efficiency microwave absorption and multifunctionality. Thanks to proper impedance matching, abundant polarization, the synergistic effect of dielectric loss and magnetic loss, as well as multiple scattering and reflection, Ni/MXene-MF exhibits strong microwave absorption (RL_min_ of -62.7 dB) corresponding to EAB of 6.24 GHz at 2 mm, and 6.88 GHz at 1.8 mm, covering the entire Ku band. In addition, good flame retardant, heat insulation, and infrared stealth functions make Ni/MXene-MF have broad application prospects in complex environments. This work provided a novel strategy for the development of multifunctional microwave absorbing materials that meet practical applications.

## Experiment Section

### Materials

Ti_3_AlC_2_ MAX powders were obtained by 11 Technology Co. MF was provided by Chengdu Yulong Chaoju New Material Co. Ltd. Lithium fluoride (LiF), Tris(hydroxymethyl)aminomethane, dopamine hydrochloride, ethylene glycol (EG), nickel chloride hexahydrate (NiCl_2_·6H_2_O), sodium hydroxide (NaOH), and ethanol were purchased from Shanghai Aladdin Co., Ltd. Hydrogen chloride (HCl) was obtained from Sinopharm Chemical Reagent Co.

### Synthesis of Ti_3_C_2_T_x_ MXene

First, 2 g of LiF was added into 40 mL of 9 M HCL and stirred for 30 min until the LiF powder was dissolved. Then, 2 g of Ti_3_AlC powder was slowly added to the mixed solution, the reaction temperature was controlled at 35 °C, and the stirring was continued for 24 h. Then, the obtained suspension was washed several times with deionized (DI) water until its pH reached 6, and then ethanol (the effect of intercalant) was added to ultrasonic centrifugation. Finally, the upper dispersion was collected after ultrasonic centrifugation with DI water and freeze-dried to obtain MXene sheets.

### Synthesis of Ni Flower

Ni flower were prepared via a facile hydrothermal process. Firstly, 1.18 g of NiCl_2_·6H_2_O and 3.6 g of NaOH were dissolved into 200 mL of EG. Then, it is stirred for 60 min until a uniform light green solution was formed. Finally, the precursor liquid was poured into the hydrothermal reactor and reacted at 200 °C for 10 h. After washing the black precipitate with deionized ethanol and water several times, dry the collected Ni flower in a vacuum drying oven for 12 h.

### Preparation of Ni Flower/MXene Hybrids

Ni flower/MXene hybrid is prepared by electrostatic self-assembly process. First, 30 mg of Ni flower was added to the CTAB solution (2 mg mL^−1^), and ultrasound for 1 h to make CTAB adsorb to the surface of Ni flower. Then, the Ni flower modified by centrifugation was washed by adding deionized water to remove excess CTAB. Finally, add the positively charged Ni flower to a few-layered MXene solutions (Ni flower: MXene = 1: 2). The prepared mixture used a shaker to shake for 24 h to ensure that the positively charged Ni flower and the negatively charged MXene are fully assembled electrostatically. Finally, the Ni flower/MXene hybrid was collected by centrifugation process and dried in a freeze dryer.

### Preparation of Ni/MXene-MF

First, the MF was ultrasonically cleaned several times with deionized water and absolute ethanol, and it was dried and ready for use. First, the surface of MF was modified by PDA (the modification method in Supporting information) to increase the surface adhesion of MF. The modified MF was immersed in 2 mg mL^−1^ Ni flower, MXene, and Ni flower/MXene suspensions for 1 min, respectively, then dried at 80 °C to remove excess water, and the above dip-coating process was repeated three times.

### Characterization

The crystal structure was identified by XRD (Bruker D8 Advance XRD) using Cu K radiation (1.5604 Å). The surface chemical compositions were analyzed by X-ray photoelectron spectroscopy (XPS, Thermo Escalab 250Xi X). The microscopic morphology and structure were observed using SEM (SEM, Zeiss Sigma 300) and transmission electron microscopy (TEM, FEI Tecnai G2 F20). The magnetic properties of samples were measured by a vibrating sample magnetometer (VSM, 7404, LakeShore, USA) at room temperature. The chemical structure was characterized by Fourier transform infrared spectroscopy (FTIR, Nicolet 6700). The infrared radiation intensity of the composite foam was measured by a thermal imaging camera (E60, FLIR). To measure the EM absorption performance, the obtained 20 wt% composites were mixed with 80 wt% paraffin were cut into concentric rings with an inner diameter of 3.04 mm and outer diameter of 7.00 mm. Then, EM parameters were measured with the coaxial line method by a vector network analyzer (VNA; Agilent 5324A).

## Results and Discussion

### Chemical Structure and Morphology

**Fig. 1:**
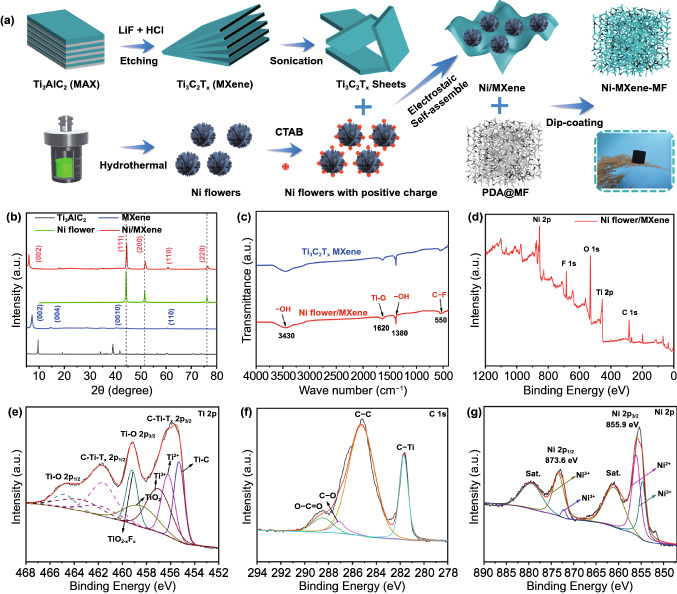
**a** Schematic illustration of preparation process for Ni/MXene-MF. **b** XRD patterns of Ti_3_AlC_2_, Ti_3_C_2_T_x_, Ni flower, and Ni/MXene hybrid. **C** FTIR spectra of Ti_3_C_2_T_x_ and Ni/MXene hybrid. The XPS of spectra of **d** Ni/MXene, **e** Ti 2p, **f** C 1s, and **g** Ni 2p

The detailed fabrication process of Ni/MXene-MF is shown in Fig. 1a. First, Ti_3_AlC_2_ was etched by LiF-HCL to remove Al atomic layers, and then the accordion-like MXene was ultrasonically processed to obtain a few-layered MXene. Due to its abundant functional groups, the obtained MXene flakes exhibit negative zeta potential [32]. Secondly, a positive charged Ni flower with good dispersibility was synthesized through hydrothermal reaction and CTAB modification. Then, the positively charged Ni was added to the negatively charged MXene. During the shaking process, the Ni was anchored on the surface of MXene through electrostatic interaction. Finally, the PDA-modified MF was immersed into Ni/MXene solution. During dip-coating processes, the Ni/MXene hybrids were assembled on the surface of MF through the capillary force of MF and the adhesive power of PDA. The Ni/MXene-MF was obtained after drying to remove excess water.

The XRD patterns of Ti_3_AlC_2_, MXene, Ni flower, and Ni/MXene hybrid are shown in Fig. 1b. The disappearance of the diffraction peak of Ti_3_C_2_T_x_ MXene at 39° indicated the Al atomic layers were removed [33]. Meanwhile, the (002) diffraction perk shifted to a lower angle from 9.7° to 7.6°, which confirmed the widen *d*-spacing of Ti_3_C_2_T_x_ MXene sheets. The observed peaks at 44.7°, 51.8°, and 76.8° in the XRD pattern of Ni were perfectly indexed to (111), (200), and (200) crystal faces of cubic crystalloid structure (PDF #03–1051) [34]. For the Ni/MXene hybrid, the typical diffraction peaks of Ni and MXene could be obviously observed, indicating the successful fabrication of the Ni/MXene hybrid. Besides, the (002) surface peak belonging to MXene in the XRD pattern of the Ni/MXene hybrid was shifted to a lower angle from 7.6° to 6.1°, indicating that the MXene layer d-spacing increased, which could be attributed to the insertion of Ni particles or the successful peeling of the few-layered MXene during the electrostatic assembly process.

The XPS was used to prove element composition and valence states information. It revealed that MXene contains Ti, C, O, and F elements (Fig. S1), while Ni/MXene mainly contains Ni, Ti, C, O, and F elements in the spectrum, verifying the combination between Ni flower and MXene sheets (Fig. 1d). The Ti 2p related four peaks of Ni/MXene at 465.1, 461.6, 459.8, and 455.7 eV correspond to Ti–O 2p_1/2_ bonds, C-Ti-T_x_ 2p_1/2_, Ti–O 2p_3/2_, and C-Ti-T_x_ 2p_3/2_, respectively (Fig. 1e) [35]. In addition, four obvious peaks found in the C 1s spectrum at 289.1, 286.5, 285.1, and 281.8 eV correspond to O-C = O, C-O, C = C, and C-Ti-T_x_ bonds, respectively (Fig. 1f) [36]. The Ni 2p spectrum included two main peaks at 873.6 and 855.9 eV, which correspond to Ni 2p_1/2_ and Ni 2p_3/2_, indicating that Ni flower was successfully introduced [22]. In addition, FTIR spectroscopy also confirmed the existence of functional groups similar to MXene in the Ni/MXene hybrid, the peaks at 550, 1620, and 3430 cm^−1^ correspond to C-F, Ti–O, and -OH bonds (Fig. 1c) [37, 38]. These abundant functional groups could cause dipole and defect polarization, thereby enhancing the EM attenuation ability.

**Fig. 2:**
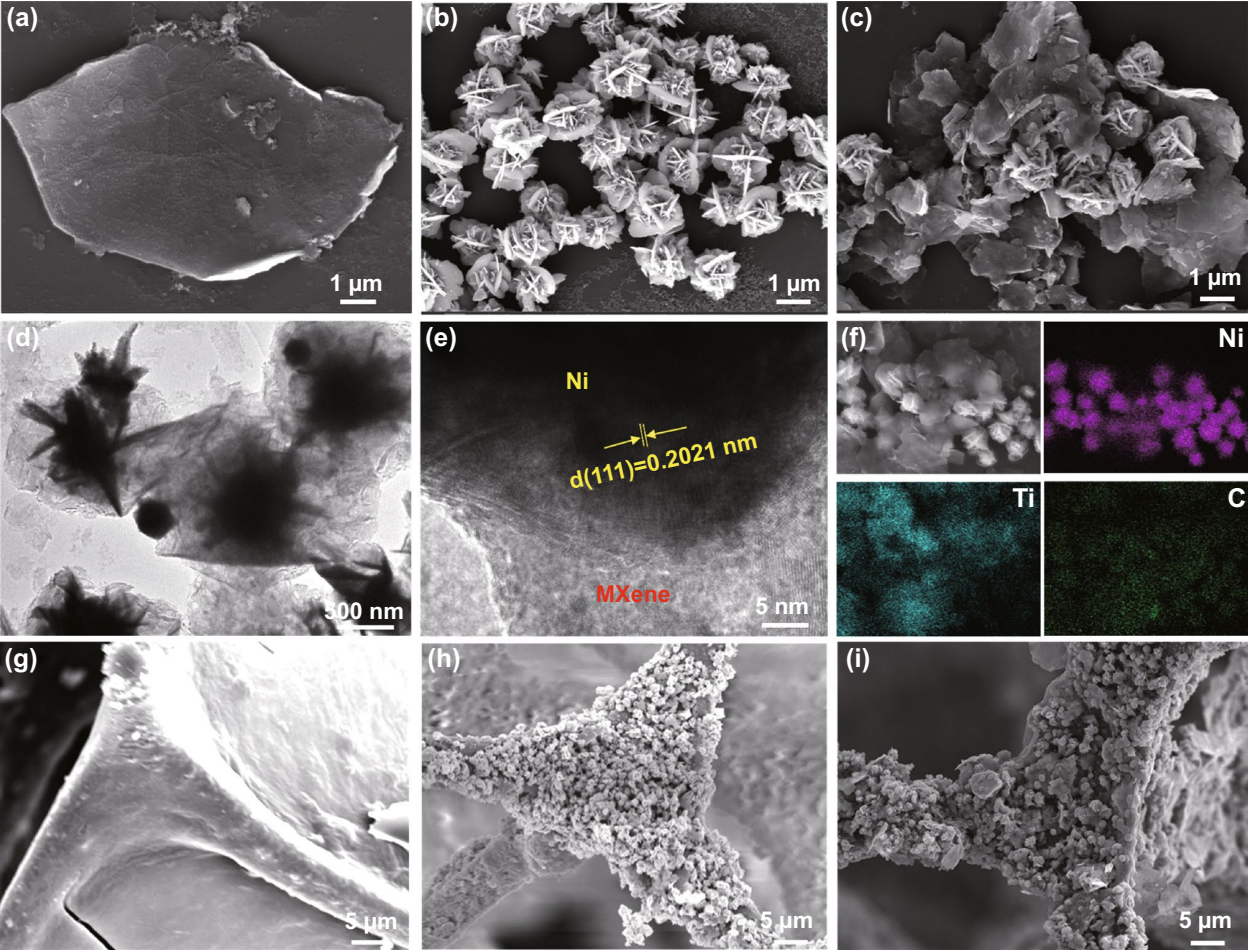
SEM images of **a** Ti_3_C_2_T_x_, **b** Ni flower, **c** Ni/MXene hybrids, **g** MXene-MF, **h** Ni flower-MF, and **i** Ni/MXene-MF. **d** TEM, **e** HR-TEM images and **f** the corresponding elemental mapping images of Ni/MXene hybrid

The morphologies of the few-layered MXene, Ni flower, and Ni/MXene hybrid were observed through the SEM and TEM. The few-layered MXene was observed a wrinkled film-like structure (Fig. 2a). Ni flower possessed a distinct petal-like structure, and its average size is about 1 μm (Fig. 2b). As for the Ni/MXene hybrid, the Ni flowers were evenly anchored on the surface of MXene or wrapped by MXene (Fig. 2c-d). The HR-TEM image of the Ni/MXene hybrid further revealed the interface and crystal structure, the interface lattice between Ni flower and MXene indicated that good coalescence occurred at the atomic level. In addition, the d-spacing (0.2021) of periodic lattice fringes corresponded to the crystal Ni with face-centered cubic (111) (Fig. 2e), which is consistent with the XRD results. The element mapping image (Fig. 2f) of the Ni/MXene hybrid confirmed the uniform distribution of Ni, Ti, and C. The synthesized MXene, Ni flower, and Ni/MXene hybrid were assembled on the surface of MF through the dip-coating adsorption process. Compared with the smooth surface of the MF, the MXene-MF skeleton and the surrounding area have a few-layered MXene successfully assembled (Fig. 2g), and the Ni flower-MF skeleton has a large number of uniform and dense particles (Fig. 2h). For the Ni/MXene-MF, the Ni/MXene hybrid wrapped on the MF skeleton tightly and homogeneously (Fig. 2i). Among them, through the electrostatic self-assembly process, the magnetic Ni flower modified on the surface of MXene could produce a large number of interfaces and form a “micro-capacitor” structure, which was conducive to improving impedance matching and generating interface polarization. In addition, the three-dimensional conductive network formed by the Ni/MXene hybrid along the MF framework help to increase the electron transmission rate and enhance the conductance loss. The porous structure of MF is conducted to EM waves multiple reflect, scatter, and absorb.

### Microwave Absorption

**Fig. 3:**
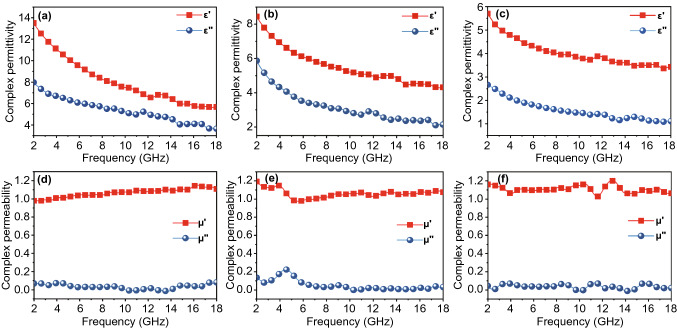
Electromagnetic parameters (*ε′*, *ε′′*, *μ′* and *μ′′*) for **a**, **d** MXene-MF, **b**, **e** Ni/MXene-MF and **c**, **f** Ni flower-MF

The EM parameters are the key to microwave absorption performance, the complex permittivity (*ε*_*r*_ = *ε′* − *jε′′*), and the complex permeability (*μ*_*r*_ = *μ′* − *jμ′′*). The real parts *ε′* and *μ′* represent the storage capacity of EM wave energy, and the imaginary parts *ε′′* and *μ′′* represent the dissipation ability [39, 40]. The EM parameters of MXene-MF, Ni/MXene-MF, and Ni-MF frequency-dependent are under the same filler loading ratio, as shown in Fig. 3. It is found that the permittivity decreased as the frequency increased, which can be attributed to the frequency dispersion effect. Among them, MXene-MF exhibited a high complex dielectric constant due to the ultra-high conductivity of MXene, the values of *ε′* and *ε′′* declined from 13.5 to 5.7 and 7.9 to 3.6 in the frequency range of 2–18 GHz, respectively. As for Ni flower-MF, the values of *ε′* and *ε′′* were low, declined from 5.7 to 3.4 and 2.6 to 1.1, respectively, indicating that Ni flower/MF possessed poor storage and dissipation capabilities of EM energy. After the electrostatic self-assembly of Ni flower and MXene, the *ε′* and *ε′′* values of the Ni/MXene-MF obtained were 8.4 to 4.3 and 5.9 to 2.1, respectively. It was worth noting that the *ε′′* value of Ni/MXene-MF was smaller than that of MXene-MF. Generally, according to the classic free electron theory ($\varepsilon^{^{\prime\prime}} = \user2{ }\frac{{\varepsilon_{s} - \varepsilon_{\infty } }}{{1 + 2\pi f^{2} \tau^{2} }}2\pi f\tau + \frac{\sigma }{{2\pi f\varepsilon_{0} }}$), the value of *ε"* increased as the value of conductivity increased [41]. Therefore, compared with MXene-MF, due to the introduction of non-conductive Ni flower and the reduction in MXene content, the conductivity (σ) of Ni/MXene-MF decreased, resulting in a decrease in *ε"* value. The appropriate *ε"*, *ε′* and σ of Ni/MXene-MF were conducive to better impedance matching and could enhance the EM absorption capacity.

Generally, the complex permeability was described as: $\mu^{^{\prime}} = 1 + \frac{M}{H}\cos \theta$ and $\mu^{^{\prime\prime}} = 1 + \frac{M}{H}\sin \theta$. Here, M, H, and θ were magnetization, external magnetic field, and phase lag angle of M behind H, respectively [42]. Due to the non-magnetic nature of MXene, the *μ′* value of MXene-MF was around 1.05, and the *μ′′* value was around 0. However, the values of *μ′* and *μ′′* of Ni/MXene-MF and Ni flower-MF were slightly increased due to the presence of magnetic Ni flower. Among them, the saturated magnetization values of Ni/MXene-MF and Ni flower-MF were 30.8 emu g^−1^ and 18.5 emu/g, respectively (Fig. S3a). Generally, the eddy current effect could be evaluated according to the C_0_ value [43]. The calculation formula of the C_0_ value was as follows:

1 $$C_{0} = \mu ^{\prime\prime}\left( {\mu ^{\prime}} \right)^{ - 2} f^{ - 1}$$

If the value of C_0_ remained constant over the entire frequency range, eddy current loss was the only source of magnetic loss [44]. As shown in Fig. S3b, the C_0_ value of Ni/MXene-MF and Ni flower-MF fluctuates with frequency, indicating that the magnetic loss mainly includes natural resonance and exchange resonance.

Since N Ni/MXene-MF possessed numerous heterogeneous interfaces and abundant functional groups, the dielectric loss mechanism was explored. The dielectric loss usually included polarization loss and conduction loss. The polarization loss could be divided into interface polarization and dipolar polarization. According to the Debye relaxation model, the type of dielectric loss could be described by the relationship between *ε′* and *ε′′*. The calculation equation is as follows [45]:

2 $$\left( {\varepsilon^{\prime} - \frac{{\varepsilon_{s} + \varepsilon_{\infty } }}{2}} \right)^{2} + \left( {\varepsilon^{\prime\prime}} \right)^{2} = \left( { \frac{{\varepsilon_{s} - \varepsilon_{\infty } }}{2}} \right)^{2}$$

Among them, *ε*_*s*_ and *ε*_*∞*_, respectively, represent static dielectric constant and relative dielectric constant. Usually, a Cole–Cole semicircle in the above formula corresponds to a Debye relaxation [46]. As shown in Fig. S4a–c, there are several small semicircles and one long tail in all three types of foams, indicating the coexistence of polarization loss and conduction loss. Specifically, multiple heterogeneous interfaces between Ni flower, MXene, MF, and air promote interfacial polarization, a large number of functional groups and lattice defects in MXene cause dipole polarization, and the MF skeleton provides a transport path for electron motion, causing conductive loss.

**Fig. 4:**
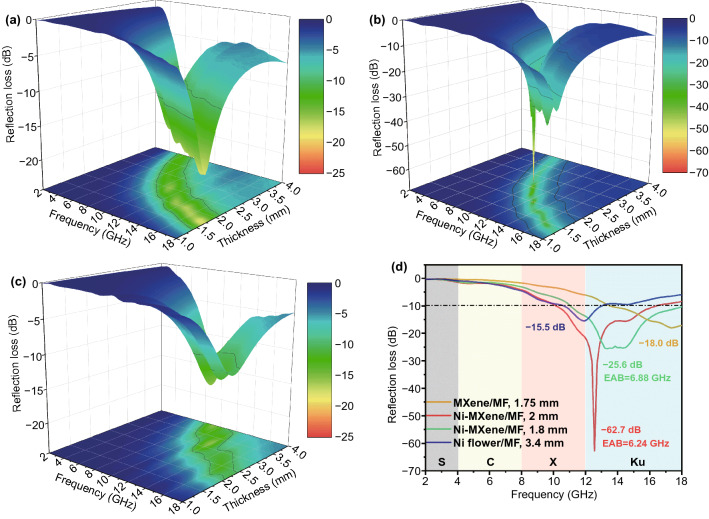
Three-dimensional representations of RL values for **a** MXene-MF, **b** Ni/MXene-MF, and **c** Ni flower-MF. **d** Frequency-dependent RL values of MXene-MF-1.75 mm, Ni/MXene-MF-2 mm, Ni/MXene-MF-1.8 mm, and Ni flower-MF-3.4 mm

EM absorption performance could be intuitively evaluated by reflection loss (RL), and the RL was calculated according to the transmission line theory as:

3 $$RL = 20\left| {\frac{{Z_{in } - Z_{0} }}{{Z_{in } +_{{Z_{0} }} }}} \right|$$

4 $$Z_{{in~}} = ~Z_{0} \sqrt {\frac{r}{r}} {\text{tanh}}\left( {j\frac{{2fd}}{c}\sqrt {rr} } \right)$$

where Z_in_ is the normalized input impedance of the absorber, Z_0_ is the impedance of free space, *f* is the frequency of the EM wave, d is the thickness of the absorber, and c is the velocity of light in free space [9, 47]. Specifically, the RL value should be lower than -10 dB, and more than 90% of the incident EM waves will be attenuated [48]. Simultaneously, to visually reveal the relationship between RL value, frequency, and thickness, the 3D reflection loss diagrams of three different samples are shown in Fig. 4a-c. It could be seen intuitively that the RL value of MXene-MF, Ni/MXene-MF, and Ni flower-MF could be adjusted by changing the thickness (Fig. S5). However, a low RL value of MXene-MF and Ni flower-MF in the thickness range of 1–4 mm, and the adjustable thickness was limited, indicating that they were not suitable for practical applications of microwave absorption. In contrast, Ni/MXene-MF exhibited outstanding microwave absorption performance. In particular, a strong RLmin of 62.7 dB with the EAB of 6.24 GHz at the ultrathin thickness of 2 mm was obtained. In addition, the EAB of the Ni/MXene-MF could reach 6.88 GHz with a thickness of 1.8 mm, which was from 11.12 to 18 GHz, as shown in Fig. 4d. Meanwhile, the relation between the absorber thickness (*t*_m_) and the peak frequency (*f*_m_) could be described by the quarter-wavelength (1/4 λ) model as Eq. 5:

5 $$t_{m} = { }\frac{{n{\uplambda }}}{4} = { }\frac{nc}{{4f_{m} \sqrt {\left| {\varepsilon_{r} } \right|\left| {\mu_{r} } \right|} }}{ }\left( {n = 1,{ }3,{ }5,{ } \ldots } \right)$$

The Ni/MXene-MF simulated curves of the RL peak frequency and the matching thickness well fits the quarter-wavelength matching conditions (Fig. S6). The results show that Ni/MXene-MF possessed excellent EM wave absorption ability, and could be expected to exhibit great practical applications in the ongoing 5G communication technology.

Generally, impedance matching and attenuation were the basic factors affecting EM wave absorption performance [49]. Three processes occurred when EM waves were incident on the surface of the material: reflection, absorption, and transmission [50]. The adjustment of impedance matching could achieve reducing reflection and increasing absorption of incident EM wave. Here, the impedance matching was evaluated by introducing the |Z_in_/Z_0_| value, which can be calculated based on Eq. 5:

6 $$\frac{{Z_{{in~}} }}{{Z_{{0~}} }} = \sqrt {\frac{r}{r}} \tanh \left( {j\frac{{2fd}}{c}\sqrt {rr} } \right)$$

**Fig. 5:**
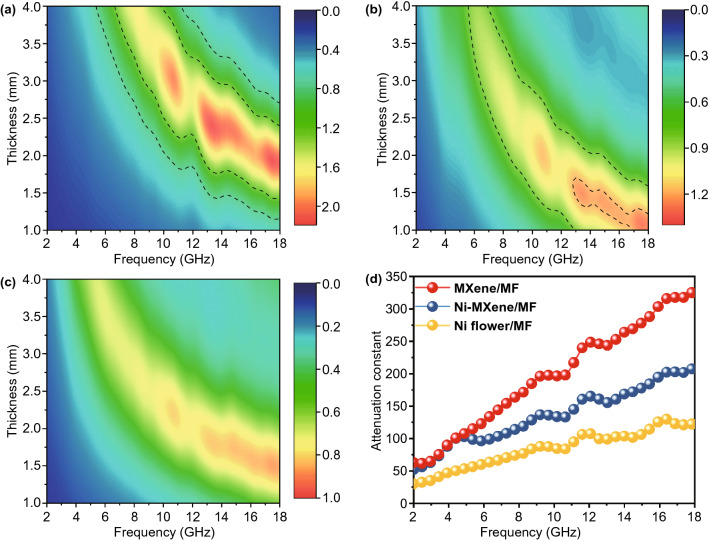
Impedance matching for **a** MXene-MF, **b** Ni/MXene-MF, and **c** Ni flower-MF. **d** The frequency dependence of the attenuation constant (α)

The value of |Z_in_/Z_0_| should be equal or close to 1 (0.8–1.2), a large amount of EM wave could enter the absorber and be attenuated [51]. The 2D contour maps of the |Z_in_/Z_0_| value of MXene-MF, Ni/MXene-MF, and Ni flower-MF are shown in Fig. 5a–c, and the values close to 1 are marked with black lines. Among them, the |Z_in_/Z_0_| value of MXene-MF was much higher than 1, and the |Z_in_/Z_0_| value of Ni flower-MF was much lower than 1, which lead to impedance mismatch, the large reflection of incident EM waves, and poor microwave absorption performance. By contrast, the black line of Ni/MXene-MF covered a broader frequency and wider thickness, and the |Z_in_/Z_0_| value was close to 1, achieving good impedance matching. The impedance matching was related to EM parameters, which were determined by the composition and structure of the absorber. Here, the impedance matching of Ni/MXene-MF was related to its porous structure, the electrical conductivity from MXene, and the magnetic permeability of the Ni flower. In addition, the absorbed incident EM wave will be dissipated or converted into other energies. Here, the microwave attenuation capability of the sample was evaluated by introducing the attenuation constant (α), and the calculation formula is as follows [52]:

7 $$\alpha = \frac{\sqrt 2 }{{\text{c}}}\pi f \times \sqrt {\left( {\mu^{^{\prime\prime}} \varepsilon^{^{\prime\prime}} - \mu ^{^{\prime}} \varepsilon^{^{\prime}} { }} \right) + \sqrt {\left( {\mu^{^{\prime\prime}} \varepsilon^{^{\prime\prime}} - \mu ^{^{\prime}} \varepsilon^{^{\prime}} { }} \right)^{2} + \left( {\mu^{^{\prime}} \varepsilon^{^{\prime\prime}} + \mu^{^{\prime\prime}} \varepsilon^{^{\prime}} { }} \right)^{2} } }$$

Figure 5d shows that the value of α gradually increased with increasing frequency. In particular, the α value changes in sequentially of Ni-MF (30.1–122.2) < Ni/MXene-MF (51.9–208.9) < MXene-MF (62.9–326.7). Based on Eq. 4, α was determined by the dielectric/magnetic loss capability. It could be seen that tanδ_*ε*_ was significantly higher than tanδ_*μ*_ (Fig. S7), which indicated that the dielectric loss has a greater effect on microwave attenuation than magnetic loss. However, the microwave absorption performance of the MXene/MF with the largest α value was not satisfactory, which shows that the attenuation ability cannot solely determine the microwave absorption of the absorbing material.

**Fig. 6:**
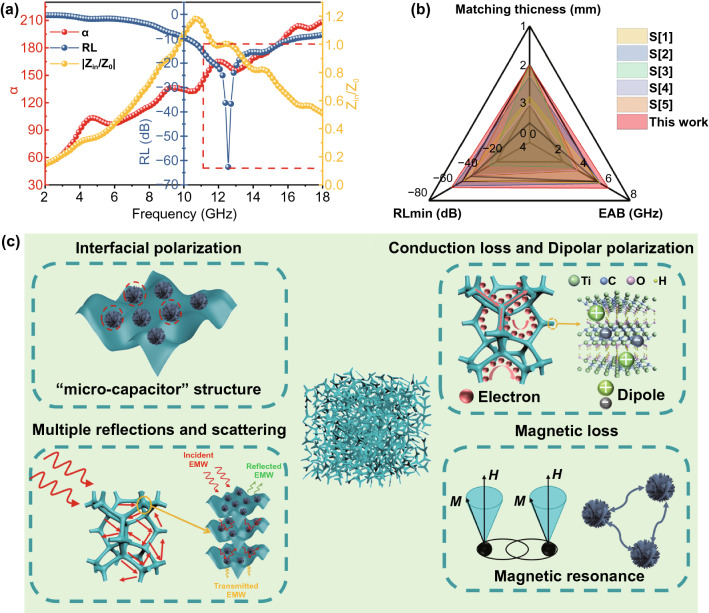
**a** Frequency-dependent α, RL, and |Z_in_/Z_0_| values of the Ni/MXene-MF-2 mm. **b** Comparison with the MA properties of the reported MF-based foam composites. **c** Schematic illustration of the microwave absorption mechanism of Ni/MXene-MF

To further reveal the potential relationship among microwave absorption performance, impedance matching and attenuation capabilities, the frequency-dependent RL value, |Z_in_/Z_0_|, and α of Ni-MXene-MF are discussed and shown in Fig. 6a. The RL value did not achieve the minimum value as the α value increased to the maximum value of 208.9. As the |Z_in_/Z_0_| value was close to 1, the RL achieved the minimum value of − 62.7 dB at the frequency of 12.56 GHz. The above results show that Ni/MXene-MF achieved excellent microwave absorption properties through balancing impedance matching and attenuation efficiency. In addition, Fig. 6b and Table S1 list the microwave absorption properties of related MF-based composites previously reported. Compared with other foam-based composite materials, Ni/MXene-MF possessed the advantages of a lightweight, thin matching (2 mm), wide EAB (6.24 GHz), and strong absorption strength (− 62.7 dB), and was regarded as an ideal choice for the EM wave absorbing materials.

### Microwave Absorption Mechanism

The microwave absorption mechanism of Ni/MXene-MF is shown in Fig. 6c. Firstly, the 3D porous framework of Ni/MXene-MF could form an interconnected conductive network, which could effectively help the migration of electrons, resulting in conduction losses and attenuating microwave energy [53–55]. Second, the Ni/MXene hybrid with a capacitor-like structure could provide a large number of interfaces, which could cause charge accumulation in the constantly changing EM field, generating interface polarization effects [56, 57]. In addition, abundant terminating functional groups and inherent defects of layered MXene will cause dipole polarization and defect polarization under high-frequency EM fields. Third, under the action of alternating EM fields, the Ni flower attenuated EM waves through natural resonance and exchange resonance [58–67]. Finally, the 3D porous network structure could increase the propagation path of microwaves in the foam, and the microwaves absorption efficiency could be improved after multiple reflection and scattering [68], while the microwaves also reflect and scatter between MXene and Ni flower.

### Multifunction

**Fig. 7:**
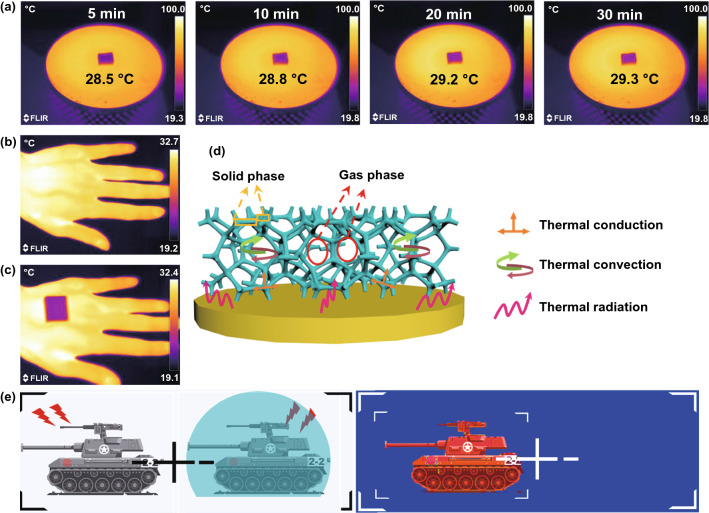
Thermal infrared images of the Ni/MXene-MF placed **a** on the heating platform at different time under 80 °C, and **b-c** on the hand. The schematic diagram of **d** the heat transfer mechanism of Ni/MXene-MF, and **e** infrared stealth application

To meet the increasing application requirements in complex environments, advanced absorbing materials need to possess multifunction, including heat insulation, infrared shielding performance, and flame retardancy [69–72]. Excellent heat insulation was an important function of absorbing materials, which could effectively protect electronic equipment or military weapon materials from overheating/overcooling temperature damage. The Ni/MXene-MF with a thickness of 1 cm was placed on a circular heating table platform at 80 °C. Meanwhile, the infrared thermal image of Ni/MXene-MF captured from 5 to 30 min is shown in Fig. 7a. The detection temperature on the upper surface of the sample was only ~ 28.5, ~ 28.8, ~ 29.2, and 29.3 °C, respectively. It is shown that Ni/MXene-MF possesses excellent heat insulation and could effectively prevent heat transfer from the bottom to the upper surface. In addition, with the extension of heating time, the upper surface temperature of Ni/MXene-MF was almost unchanged, indicating that it possesses excellent thermal insulation stability. More importantly, the superior heat insulation of MXene-MF made it possess an infrared stealth function, which could shield infrared radiation to hide military targets. Generally, objects with higher temperatures will show higher infrared radiation intensity under infrared thermal imaging. As shown in Fig. 7b, it could be seen that the color of the bare hand was different from the surrounding environment, making it visible under the infrared detection equipment. On the contrary, with a piece of Ni/MXene-MF was placed on the back of the hand, the color of the covered area was consistent with the color of the surrounding environment (Fig. 7c), making it disappear under the infrared detection equipment. Therefore, Ni/MXene-MF possesses strong infrared shielding properties. In addition, the flame retardancy of Ni/MXene-MF was verified through combustion experiments. As shown in Movie S1, MF possesses natural flame retardancy. When exposed to an open flame, the MF foam surface begins to burn and automatically extinguishes after leaving the flame, and without producing droplets. This is due to the MF rapidly forming dense coking on the burning surface and effectively preventing the combustion from developing to a deeper level. However, it cannot be used due to the shrinkage of the shape. On the contrary, Ni/MXene-MF maintained its original shape after burning (Movie S2). This is because the Ni/MXene hybrid was assembled on the surface of the MF to form a dense flame-retardant protective layer, and the high porosity could promote the rapid diffusion of heat during the combustion process.

The excellent heat insulation performance mechanism of Ni/MXene-MF is shown in Fig. 7d. The heat transfer methods of foam materials include heat conduction, heat convection, and heat radiation. The total thermal conductivity was related to the solid phase, gas phase, and radiation heat transfer, respectively [24, 73]. There is a large amount of air in the foam, and its thermal conductivity was much lower than that of the Ni/MXene-MF framework. Therefore, high porosity could significantly reduce the thermal conductivity and radiation heat transfer capacity of the solid phase. In addition, the long ligaments of MF could extend the heat conduction path, thereby reducing the heat transfer capacity. Accordingly, Ni/MXene-MF possesses excellent thermal insulation, infrared shielding, and flame retardancy, which could reduce deformation and damage caused in harsh environments, and provide a broad space for infrared stealth applications (Fig. 7e).

## Conclusions

In conclusion, the multifunctional Ni/MXene-MF was successfully prepared through electrostatic self-assembly and dip-coating process. The well-designed Ni/MXene micro-capacitor structure and 3D porous structure achieved good impedance matching and strong attenuation efficiency. Ni/MXene-MF exhibited the best EMA performance with a minimum RL of − 62.7 dB and the corresponding EAB reached 6.24 GHz with the thickness of only 2 mm. Meanwhile, the EAB reached a maximum of 6.88 GHz with a thickness of 1.8 mm. Besides, the novel Ni/MXene-MF exhibits good heat insulation, infrared stealth, and flame-retardant functions, making it a huge application potential in harsh environments. Therefore, this work provides design inspiration for multifunctional foams with efficient EM wave absorption properties.

## Supplementary Information

Below is the link to the electronic supplementary material.

Supplementary file1 (MP4 844 kb)

Supplementary file2 (MP4 689 kb)

Supplementary file3 (PDF 1226 kb)
