# Correction to: Ni Flower/MXene-Melamine Foam Derived 3D Magnetic/Conductive Networks for Ultra-Efficient Microwave Absorption and Infrared Stealth

**DOI:** 10.1007/s40820-022-00820-w

**Published:** 2022-04-28

**Authors:** Haoran Cheng, Yamin Pan, Xin Wang, Chuntai Liu, Changyu Shen, Dirk W. Schubert, Zhanhu Guo, Xianhu Liu

**Affiliations:** 1 Key Laboratory of Advanced Material Processing & Mold (Ministry of Education), National Engineering Research Center for Advanced Polymer Processing Technology, College of Materials Science and Engineering, Zhengzhou University, Zhengzhou, 450002 People’s Republic of China; 2 Institute of Polymer Materials, Friedrich-Alexander-University Erlangen-Nuremberg, Martensstr. 7, 91058 Erlangen, Germany; 3 Integrated Composites Laboratory (ICL), Department of Chemical & Biomolecular Engineering, University of Tennessee, Knoxville, TN 37996 USA

## Correction to: Nano-Micro Lett. (2022) 14:63 https://doi.org/10.1007/s40820-022-00812-w

The original version of this article unfortunately contained some mistakes. The corrections are updated as follows:

**Error 1:**

We found that Equations 3, 4 and 6 were wrong in the published paper:

3 $$ RL = 20\left| {\frac{{Z_{in } - Z_{0} }}{{Z_{in } +_{{Z_{0} }} }}} \right| $$

4 $$ Z_{in } = Z_{0} \sqrt{\frac{r}{r}}  \tanh \left( {j\frac{2\pi fd}{c}\sqrt {rr} } \right) $$

6 $$ \frac{{Z_{in } }}{{Z_{0 } }} = \sqrt{\frac{r}{r}}  \tanh \left( {j\frac{2\pi fd}{c}\sqrt {rr} } \right) $$

It should be corrected to the following formula

3 $$ RL = 20\log \left| {\frac{{Z_{in } - Z_{0} }}{{Z_{in } + Z_{0} }}} \right| $$

4 $$ Z_{in } = Z_{0} \sqrt {\frac{{\mu_{r} }}{{\varepsilon_{r} }}} \tanh \left( {j\frac{2\pi fd}{c}\sqrt {\mu_{r} \varepsilon_{r} } } \right) $$

6 $$ \frac{{Z_{in } }}{{Z_{0 } }} = \sqrt {\frac{{\mu_{r} }}{{\varepsilon_{r} }}} \tanh \left( {j\frac{2\pi fd}{c}\sqrt {\mu_{r} \varepsilon_{r} } } \right) $$

**Error 2:**

In the page 7, “Since N Ni/MXene-MF possessed numerous heterogeneous interfaces and abundant functional groups, the dielectric loss mechanism was explored.”

Should be:

“Since Ni/MXene-MF possessed numerous heterogeneous interfaces and abundant functional groups, the dielectric loss mechanism was explored.”

**Error 3:**

In the page 8, “In particular, a strong RLmin of 62.7 dB with the EAB of 6.24 GHz at the ultrathin thickness of 2 mm.”

Should be:

In the page 8, “In particular, a strong RL_min_ of − 62.7 dB with the EAB of 6.24 GHz at the ultrathin thickness of 2 mm.”

**Error 4:**

In the page 8, “Here, the impedance matching was evaluated by introducing the |Z_in_/Z_0_| value, which can be calculated based on Eq. 5”; and, in the page 9, “Based on Eq. 4”.

Should be:

“Here, the impedance matching was evaluated by introducing the |Z_in_/Z_0_| value, which can be calculated based on Eq. 6”; and “Based on Eq. 7”.

