# Supplementary material for: Ni Flower/MXene-Melamine Foam Derived 3D Magnetic/Conductive Networks for Ultra-Efficient Microwave Absorption and Infrared Stealth
Source: Nanomicro Lett. 2022 Feb 21;14:63. doi: 10.1007/s40820-022-00812-w (PMC8861240; doi:10.1007/s40820-022-00812-w)
Supplement: Supplementary file 3 — Supplementary file3 (PDF 1226 kb) [file 40820_2022_812_MOESM3_ESM.pdf]

Supporting Information for

Ni Flower/MXene-Melamine Foam Derived 3D Magnetic/Conductive Networks for Ultra-Efficient Microwave Absorption and Infrared Stealth

Haoran Cheng[1, #], Yamin Pan[1, #], Xin Wang[2], Chuntai Liu[1], Changyu Shen[1], Dirk W. Schubert[2], Zhanhu Guo[3], Xianhu Liu[1, *]

[1]College of Materials Science and Engineering, National Engineering Research Center for Advanced Polymer Processing Technology, Key Laboratory of Advanced Material Processing & Mold (Ministry of Education), Zhengzhou University, Zhengzhou, 450002, P. R. China

[2]Institute of Polymer Materials, Friedrich-Alexander-University Erlangen-Nuremberg, Martensstr. 7, 91058, Erlangen, Germany

[3] Integrated Composites Laboratory (ICL), Department of Chemical & Biomolecular Engineering, University of Tennessee, Knoxville, TN 37996 USA

[#]Haoran Cheng and Yamin Pan contributed equally to this work

*Corresponding author. E-mail: xianhu.liu@zzu.edu.cn (Xianhu Liu)

S1 Preparation of MF@PDA Foam

0.4 g Tris was dissolved in 200 ml deionized water, then adjust the pH to 8.5 with 0.5 g/mol HCl, and stir for 30 min. Then add 0.4 g of dopamine hydrochloride to the solution, and then cut the MF into the required size and put it into the above solution. After stirring for 12 h, rinse with deionized water and vacuum dry to get PDA@MF.

S2 Supplementary Figures and Tables

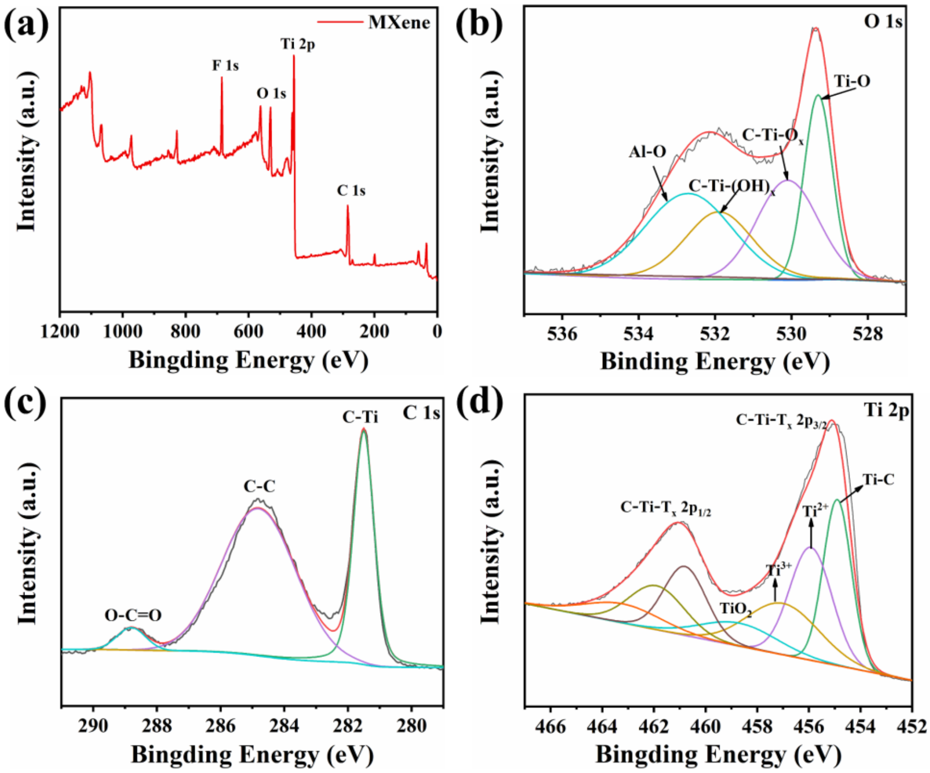

Fig. S1 (**a**) The XPS spectra of $Ti_3C_2T_x$ MXene. (**b**) O 1s spectra, (**c**) C 1s spectra, and (**d**) Ti 2p spectra

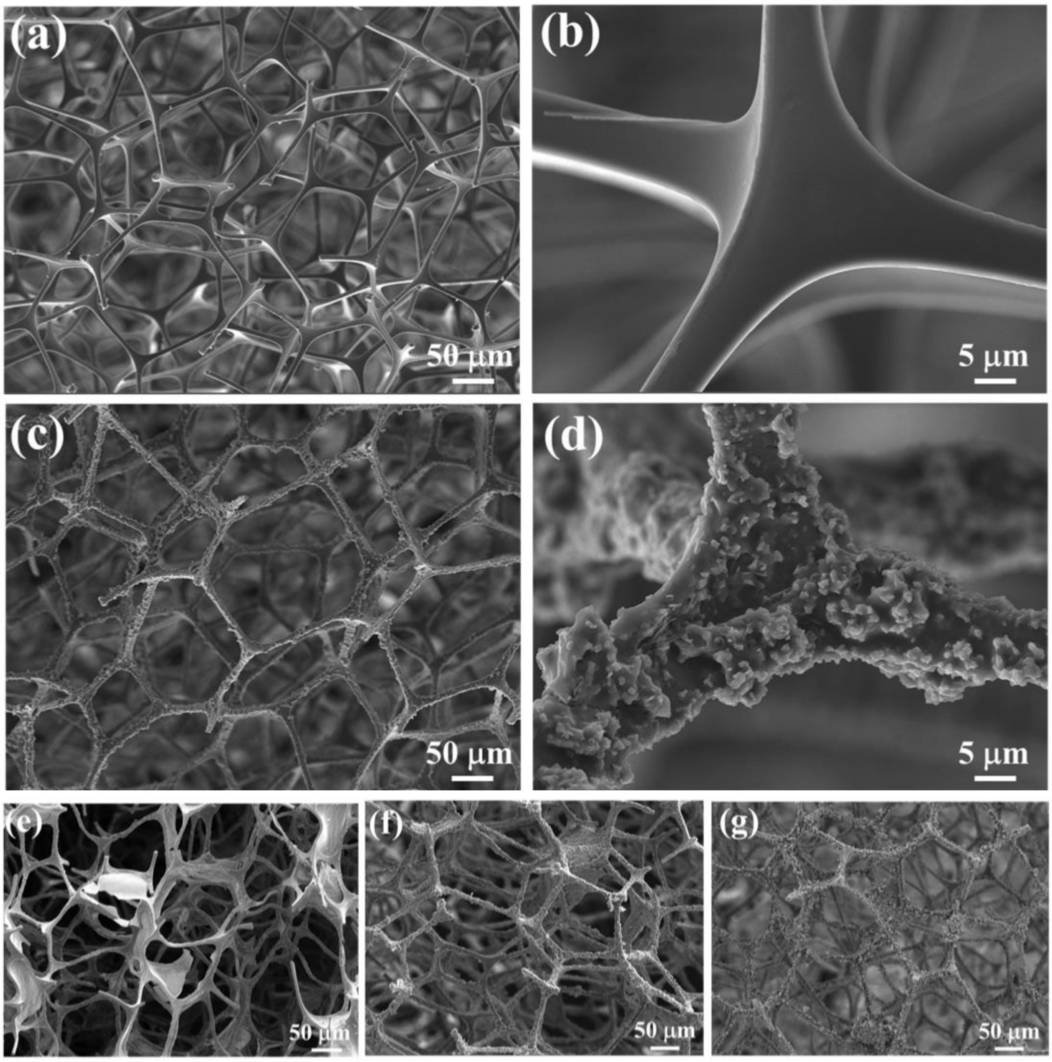

Fig. S2 SEM images of MF (**a,b**), PDA@MF(**c,d**), MXene-MF (**e**), Ni/MXene-MF, and Ni flower-MF

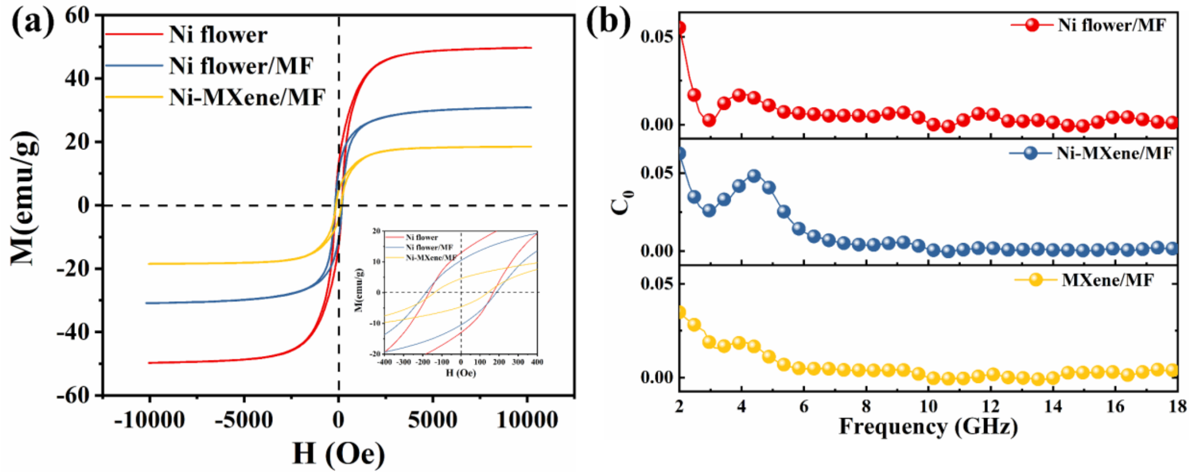

Fig. S3 (**a**) Hysteresis loops of pure Ni flower, Ni flower-MF, and Ni/MXene-MF. (**b**) C_0 values of the Ni flower-MF, Ni/MXene-MF, and MXene-MF

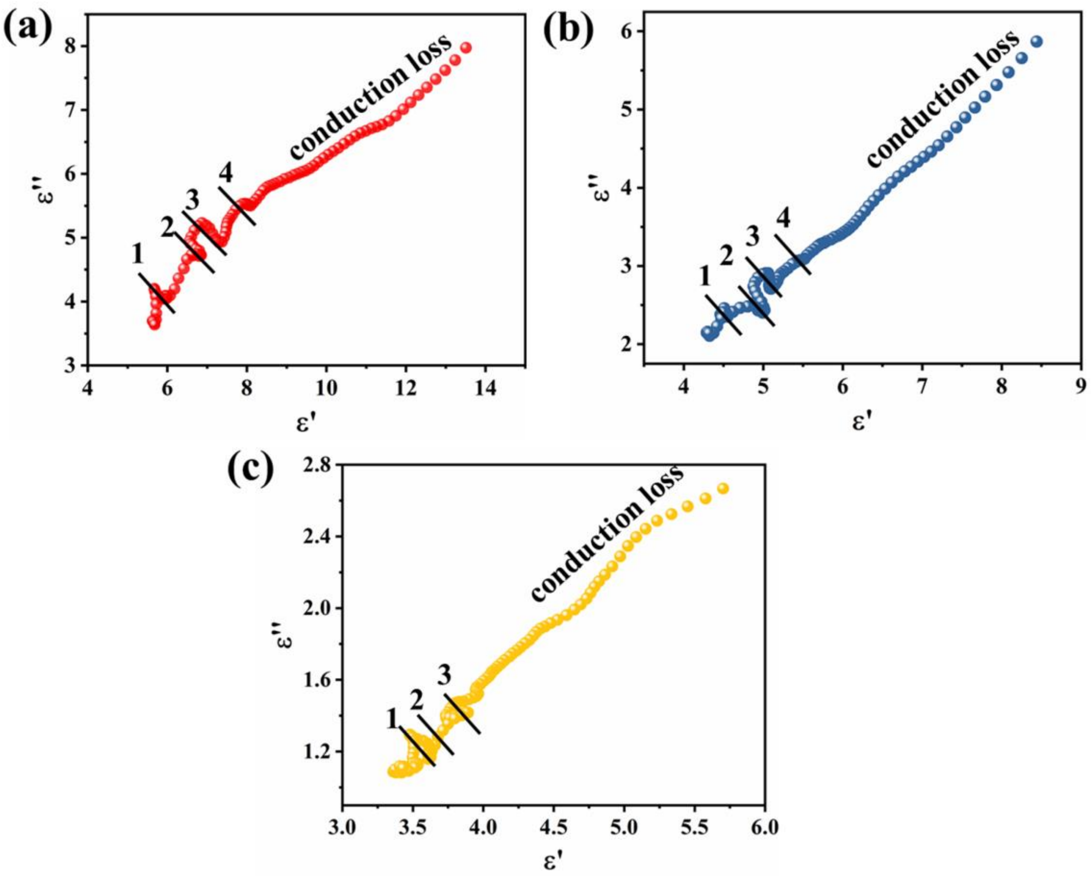

Fig. S4 Cole–Cole plots of (**a**)MXene-MF, (**b**)Ni/MXene-MF, and (**c**) Ni flower-MF

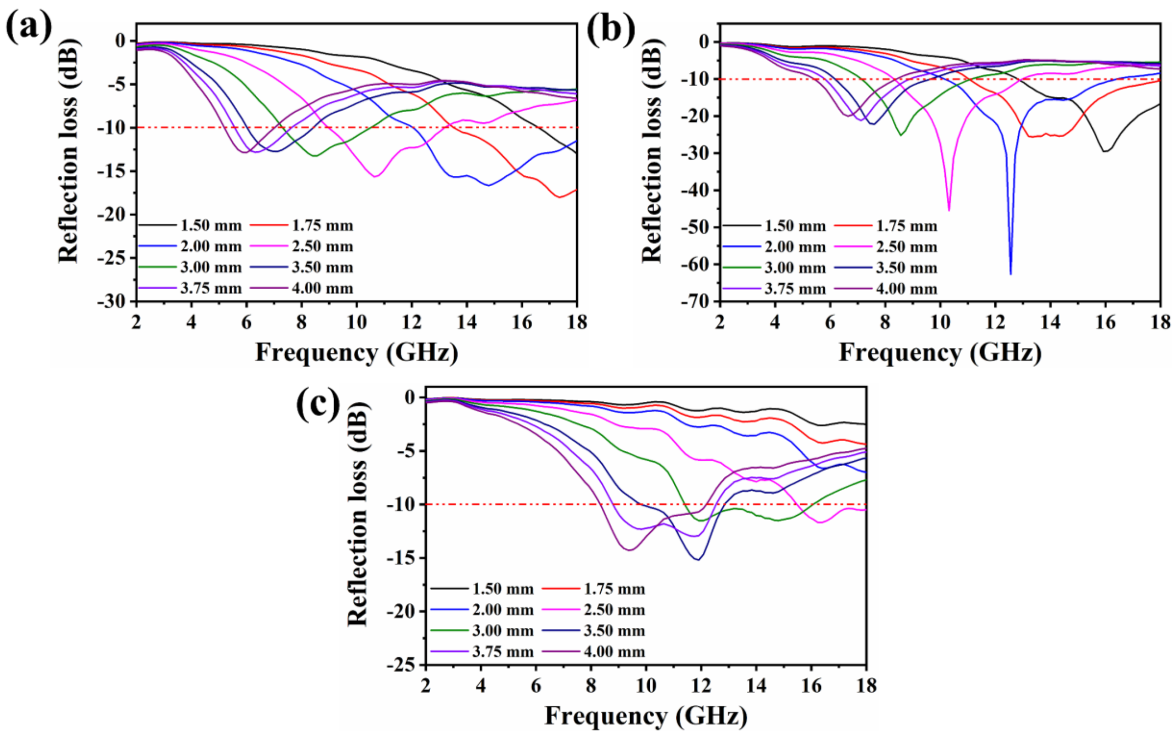

Fig. S5 RL curves with various thicknesses for (**a**) MXene-MF, (**b**)Ni/MXene-MF, and (**c**) Ni flower-MF

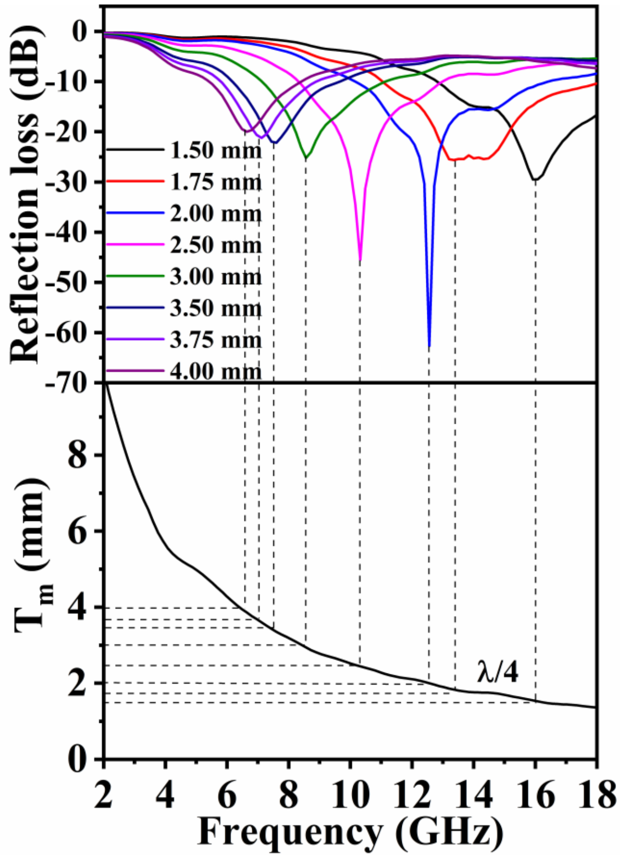

Fig. S6 Dependence of 1/4λ matching thickness on RL peak frequency for Ni/MXene-MF

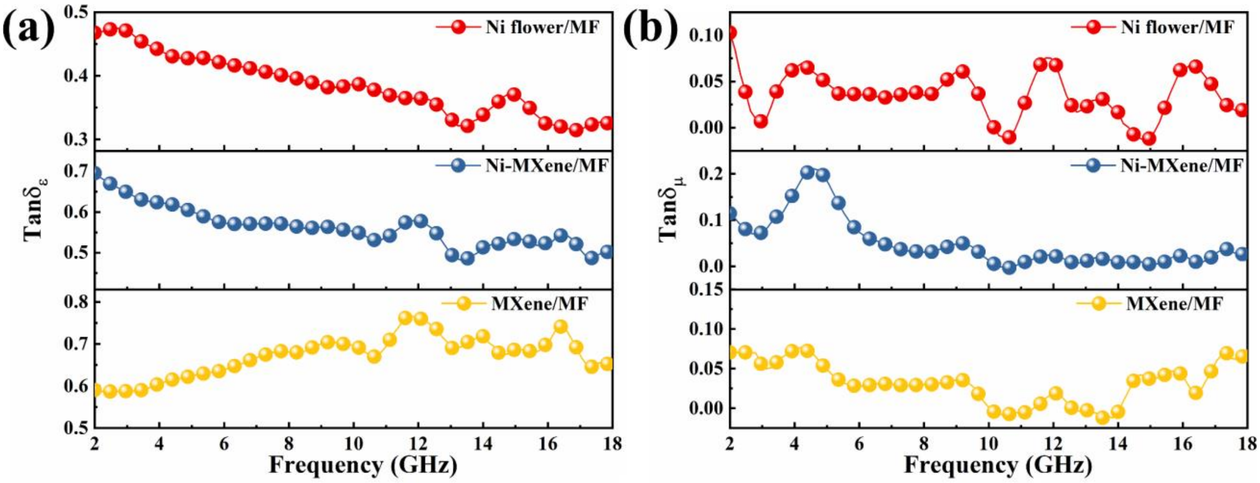

Fig. S7 Dielectric loss curves (**a**), magnetic loss curves (**b**) for MXene-MF, Ni/MXene-MF, and Ni flower-MF

Table S1 Comparison of EM wave absorption performance of related MF-based composites

Materials	Filler content	RL_{min} Value (dB)	RL< -10 dB (GHz)	Thickness (mm)	Refs
SiC_{nw}/MF	NA	-52.49	5.6	2.82	[S1]
ZIF-67/MF	20 wt%	-59.82	5.64	2.3	[S2]
Co_3O_4/RGO/MF	10 wt%	-31.88	3.4	2	[S3]

PPy@MoS_2/CMF	20 wt%	-45.4	3.8	3	[S4]
Co_3O_4 nanosheet/CMF	40 wt%	-46.58	5.4	3.5	[S5]
SiC/MF	40 wt%	-29.5	2.64	1.75	[S6]
SiC/CMF	50 wt%	-51.58	10.84	3.6	[S7]
MF@GMC	NA	-47.5	6.72	3	[S8]
Ni/MXene/MF	20 wt%	-62.7	6.24	2	This work

Table S2 Comparison of thermal insulation properties of other materials

**Materials**	**Hot platform temperature (°C)**	**Sample surface temperature (°C)**	**Refs**
AgNWs/Fe_3O_4/MF	80	32.3	[S9]
Co/CNTs/EG	63.5	28.8	[S10]
Fe/Fe_2O_3@porous carbon	62.6	29.4	[S11]
PAN/CNT/Fe_3O_4 aerogel	100	42.31	[S12]
Shaddock Peel-Based Carbon Aerogel	68.7	35.7	[S13]
Ni/MXene/MF	80	29.3	This work

Movie S1 Combustion experiment of MF

Movie S2 Combustion experiment of Ni-MXene/MF

Supplementary References

[S1] K. Su, Y. Wang, K. Hu, X. Fang, J. Yao et al., Ultralight and high-strength SiCnw@SiC foam with highly efficient microwave absorption and heat insulation properties. ACS Appl. Mater. Interfaces **13**(18), 22017-22030 (2021). https://doi.org/10.1021/acsami.1c03543

[S2] W. Gu, J. Tan, J. Chen, Z. Zhang, Y. Zhao et al., Multifunctional bulk hybrid foam for infrared stealth, thermal insulation, and microwave absorption. ACS Appl. Mater. Interfaces **12**(25), 28727-28737 (2020). https://doi.org/10.1021/acsami.0c09202

[S3] Y. Li, S. Li, T. Zhang, L. Shi, S. Liu et al., 3D hierarchical Co_3O_4/reduced grapheneoxide/melamine derived carbon foam as a comprehensive microwave absorbing material. J. Alloy. Compd. **792**, 424-431 (2019). https://doi.org/10.1016/j.jallcom.2019.03.359

[S4] Z. Yang, H. Guo, W. You, Z. Wu, L. Yang et al., Compressible and flexible PPy@MoS_2/C microwave absorption foam with strong dielectric polarization from 2D semiconductor intermediate sandwich structure. Nanoscale **13**(9), 5115-5124 (2021).

https://doi.org/10.1039/d0nr08794g

[S5] L. Lyu, S. Zheng, F. Wang, Y. Liu, J. Liu, High-performance microwave absorption of mof-derived Co_3O_4@N-doped carbon anchored on carbon foam. J. Colloid Interface Sci. **602**, 197-206 (2021). https://doi.org/10.1016/j.jcis.2021.05.184

[S6] X. Ye, Z. Chen, S. Ai, B. Hou, J. Zhang et al., Porous SiC/melamine-derived carbon foam frameworks with excellent electromagnetic wave absorbing capacity. J. Adv. Ceram. **8**(4), 479-488 (2019). https://doi.org/10.1007/s40145-019-0328-2

[S7] X. Ye, Z. Chen, M. Li, T. Wang, C. Wu et al., Microstructure and microwave absorption performance variation of SiC/C foam at different elevated-temperature heat treatment. ACS Sustain. Chem. Eng. **7**(22), 18395-18404 (2019). https://doi.org/10.1021/acssuschemeng.9b04062

[S8] T. Guo, X. Chen, G. Zeng, J. Yang, X. Huang et al., Impregnating epoxy into N-doped-CNTs@carbon aerogel to prepare high-performance microwave-absorbing composites with extra-low filler content. Compos. Part A Appl. Sci. Manuf. **140**, 106159 (2021). https://doi.org/10.1016/j.compositesa.2020.106159

[S9] H.G. Shi, H.B. Zhao, B.W. Liu, Y.Z. Wang, Multifunctional flame-retardant melamine-based hybrid foam for infrared stealth, thermal insulation, and electromagnetic interference shielding. ACS Appl. Mater. Interfaces **13**, 26505−26514 (2021). https://doi.org/10.1021/acsami.1c07363

[S10] Z. Xiang, X. Zhu, Y. Dong, X. Zhang, Y. Shi et al., Enhanced electromagnetic wave absorption of magnetic Co nanoparticles/CNTs/EG porous composites with waterproof, flame-retardant and thermal management functions. J. Mater. Chem. A **9**(32), 17538-17552 (2021). https://doi.org/10.1039/d1ta05181d

[S11] X. Zhu, Y. Dong, Z. Xiang, L. Cai, F. Pan et al., Morphology-controllable synthesis of polyurethane-derived highly cross-linked 3D networks for multifunctional and efficient electromagnetic wave absorption. Carbon **182**, 254-264 (2021). https://doi.org/10.1016/j.carbon.2021.06.028

[S12] Y. Li, X. Liu, X. Nie, W. Yang, Y. Wang et al., Multifunctional organic–inorganic hybrid aerogel for self-cleaning, heat-insulating, and highly efficient microwave absorbing material. Adv. Funct. Mater. **29**(10), 1807624 (2019). https://doi.org/10.1002/adfm.201807624

[S13] W. Gu, J. Sheng, Q. Huang, G. Wang, J. Chen et al., Environmentally friendly and multifunctional shaddock peel-based carbon aerogel for thermal-insulation and microwave absorption. Nano-Micro Lett. **13**, 102 (2021). https://doi.org/10.1007/s40820-021-00635-1
